# Inkjet-Printed MoS_2_ D‑Shaped Fiber for Resonance-Enhanced Optical Sensing

**DOI:** 10.1021/acsphotonics.6c01158

**Published:** 2026-09-30

**Authors:** Amar N. Ghosh, Mingfei Xiao, Jinrui Chen, Victor Pacheco-Peña, Meng Huang, Noel Healy, Tawfique Hasan, Anna C. Peacock

**Affiliations:** † Optoelectronics Research Center, 7423 University of Southampton, Southampton SO17 1BJ, U.K.; ‡ Department of Engineering, 2152 University of Cambridge, Cambridge CB3 0FA, U.K.; § School of Mathematics, Statistics, and Physics, Newcastle University, Newcastle Upon Tyne NE1 7RU, U.K.

**Keywords:** fiber optics, two-dimensional materials, inkjet printing, optical properties, refractive index sensor

## Abstract

Two-dimensional (2D) materials have shown great potential for applications in environmental monitoring and sensing due to their unique physical and chemical properties. In particular, molybdenum disulfide (MoS_2_) has emerged as a versatile 2D material platform for many optical and optoelectronic applications due to its high refractive index, large surface-to-volume ratio, and tunable bandgap. In this work, the integration of MoS_2_ layers on a side-polished optical fiber with a D-shaped cross-section has been demonstrated using a low-cost and scalable inkjet printing technique. The MoS_2_-integrated optical fibers are shown to offer strong wavelength-dependent light-matter interactions that can be controlled by the thickness of the 2D material, allowing for tuning of their operation across the entire telecom band. Using a printed device with a MoS_2_ layer thickness of 30 nm, designed to exhibit a resonance-enhanced transmission dip at wavelengths of ∼1.50 μm, a refractive index sensor has been demonstrated using saline solutions. The experimental results have been verified via numerical simulations, confirming the sensitivity and flexibility offered by the 2D material structures. These results indicate that inkjet-printed MoS_2_ optical fiber devices are suitable for the development of large-scale sensing networks.

## Graphical abstract

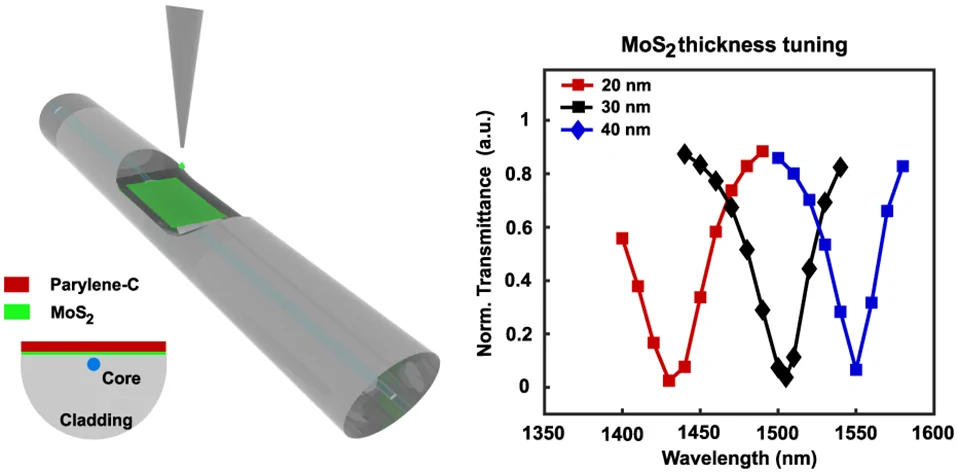

## Introduction

Real-time environmental monitoring is critical to ensuring the comfort and safety of humans. As such, there is growing interest in the development of low-cost and efficient optical sensors for the detection of gases, pathogens, and environmental pollutants in the air and water. In recent years, two-dimensional (2D) nanomaterials, including graphene and transition metal dichalcogenides (TMDCs), have been widely explored for sensor development due to their excellent optoelectronic properties and suitability for integration with a diverse range of waveguide platforms.^1–5^ TMDCs have the general formula MX_2_, with M as a transition metal and X as a group VI element such as S, Se, or Te.^6^ These materials, which include MoS_2_, have a layer-dependent band structure,^7^ making it possible to tune their optoelectronic properties through the material thickness, expanding the operational capacity of the devices. In addition, MoS_2_ offers a relatively high thermal and chemical stability, allowing for the construction of robust and durable sensors for long-term monitoring.^8^

Notably, the integration of 2D materials onto optical fiber platforms has received much interest for the development of remote or distributed sensing platforms, especially for use in harsh environments.^9^ Most of these demonstrations have used materials that have been grown or deposited using chemical vapor deposition (CVD) or metal–organic chemical vapor deposition (MOCVD) techniques^10–13^ before being transferred to the fibers.^14,15^ On the other hand, inkjet printing offers a straightforward and scalable additive manufacturing approach that allows direct deposition of 2D materials onto optical fiber platforms.^16^ Compared to methods that involve the growth and subsequent transfer of the material, printing has the advantage of positioning the functional material at precise locations anywhere along the fiber length.^17,18^

In this paper, the direct deposition of MoS_2_ materials onto D-shaped, side-polished, single-mode optical fibers (SMFs) has been demonstrated using inkjet printing. The printing method is able to precisely position the 2D material onto the polished fiber region, with good control of the layer thickness by adjusting the number of print-paths. Raman spectroscopy conducted on the printed MoS_2_ samples has confirmed the good crystallinity of the flakes that contribute to the printed layers, with spectral characteristics that are largely independent of the thickness of the printed layer.^19^ Using numerical simulations, strong wavelength-dependent light-matter interactions are confirmed to occur when the wave-guided light is coupled to the MoS_2_ layer, a process that can be tuned through the material thickness. By selecting the optimum design for operation in the telecom band, a device with 6 print-paths has been experimentally measured to exhibit a strong interaction in the wavelength region of 1.46–1.68 μm. The light-matter interaction manifests as a dip in the transmittance spectrum of ∼−60 dB, yet the average device loss remains low enough for practical use (∼3 dB). To demonstrate the application potential of the enhanced 2D material interaction, the D-shaped fiber device has been used as a refractive index sensor. The ability to accurately determine the concentration of various saline solutions is verified through experimental measurements and supported by numerical modeling of how light couples into the layered structure. We expect such devices to be of use in a wide range of environmental monitoring scenarios for the detection of both airborne and water-based contaminants.

## Experimental Section

### Fabrication of MoS_2_-Printed Devices

A schematic representation of the MoS_2_-printed optical fiber device is shown in Figure 1. The device is developed on a side-polished SMF with standard cladding and core diameters of 125 and 8.2 μm, respectively. The side-polishing process uses a custom-made metal polishing block to form a flat D-shaped region with adiabatic transitions connecting to the initial circular geometry at the input and output, as described in ref. 15. This structure thus allows the core-guided mode of the fiber to seamlessly transition into the region where it interacts with the MoS_2_ layer without breaking its optical path. The polishing block was designed to produce a uniform interaction region of length ∼1 cm on the side of the SMF, with an ultrasmooth and flat surface (a roughness of <1 nm (RMS) and a longitudinal angle of 0.04° (Supporting Information: Polished fiber surface characterization, (Figure S1)). The high surface quality of the polished region ensures negligible transmission loss in the modified SMF and good bonding with the printed layer. During the polishing process, a cladding buffer of ∼1 μm was retained above the core to prevent detrimental transmission loss in the device. This buffer thickness was determined by monitoring the transmission through the core when a high-index fluid was placed on top of the polished region. Further verification was provided by microscopy measurements to determine the width of the polished surface as compared to the cladding diameter.^15^ For the ∼1 μm polishing depth, the transmission measurements provided access to ∼50 dB of the propagating light when the liquid was applied to the surface. In addition, the polished surface was ensured to be parallel to the fiber core, which is critical to achieving efficient resonant coupling.^14^

**Figure 1:**
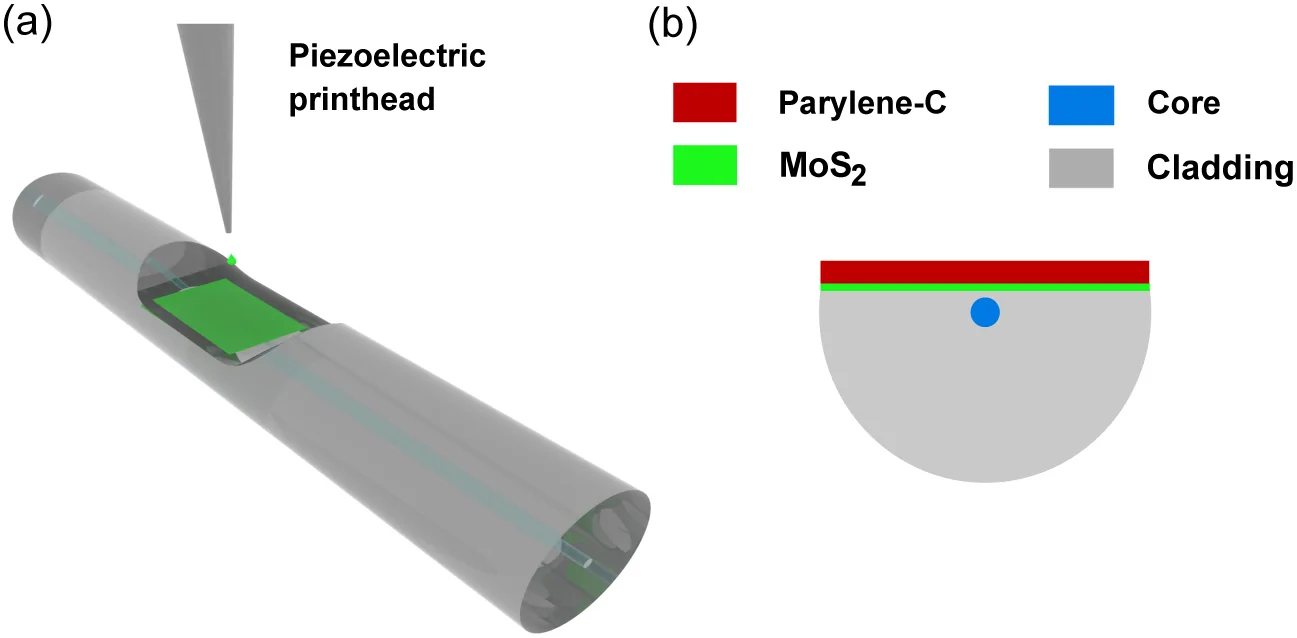
Fabrication of MoS_2_-printed fiber devices. (a) Schematic representation of the inkjet printing process, where a piezoelectric printhead is used to print the MoS_2_ onto the polished region of a D-shaped SMF. Subsequently, the parylene-C layer is deposited via chemical vapor deposition (CVD). (b) Cross-sectional view of the device showing the printed MoS_2_ and the parylene-C passivation layer on top of the SMF cladding.

Inkjet printing was then employed to print MoS_2_ layers on the polished region of the D-shaped fiber,^17,18^ as indicated in Figure 1a. Before printing, a rigorous cleaning protocol using isopropyl alcohol (IPA) and a high-pressure nitrogen blow-off was employed to clear the polished surface, ensuring pristine contact for the direct printing of MoS_2_. The ink was prepared by high-pressure homogenization (HPH) exfoliation of MoS_2_,^19^ which produces few-layer MoS_2_ flakes with ∼50 nm lateral size and ∼3 nm thickness (representing ∼4–5 layers) at a concentration of 0.5 mg/mL. The ink carrier was a binary solvent mixture of IPA with 10 vol % 2-butanol, designed to mitigate nonuniform material deposition due to the coffee ring effect during drying.^18^ The ink containing the MoS_2_ nanosheets was printed onto the polished surface of the fiber with different numbers of print-paths using a Dimatix 2850 inkjet printer equipped with a 10 pL Dimatix materials cartridge. The printing process used an optimized droplet spacing of 25 μm to promote uniform and continuous deposition. During printing, the platen temperature was maintained at 60 °C to enhance solvent evaporation and film uniformity. The printed MoS_2_ layer had a width of 124 μm and a length of 1 cm. The MoS_2_ layers were then encapsulated with a 1-μm-thick parylene-C passivation layer, as shown in Figure 1b. The parylene-C was deposited using a CVD process at room temperature (ca. 25 °C). The printed MoS_2_ flakes remained undisturbed during the process, as this deposition is entirely nondestructive and free of thermal mismatch stress. Due to the high index of the parylene-C (*n* = 1.639 across the telecom band),^20^ and this layer also helps to draw the evanescent tail of the core-propagating mode toward the 2D material to improve the light-matter interaction.^15^ However, it is worth noting that the 1 μm capping layer was sufficiently thin to ensure that the propagating mode remained bound to the core, so that no radiation loss was induced.

### Material Characterization

To characterize the properties of the inkjet-printed MoS_2_ layers, Raman spectroscopy and thickness measurements were performed. For this purpose, a series of MoS_2_ samples were prepared by printing layers with an ink concentration of 0.5 mg/mL onto microscope glass slides. It is noted that each layer is identified as one print-path. First, Raman spectroscopy (Renishaw inVia) was used to determine the quality of the printed MoS_2_ layers, where a 532 nm Nd:YAG laser was focused to a spot diameter of 1 μm on the MoS_2_ samples using a 50 × microscope objective (NA= 0.75). Measurements were taken using a confocal microscope with a diffraction grating of 2400 lines/mm and a precision of 0.12 cm^–1^, using low excitation powers to avoid unintentional heating of the sample. The Raman spectra of three different MoS_2_ samples with 2, 8, and 20 print-paths, measured with the same incident power, are shown in Figure 2a. All three spectra show the two characteristic *E*_2g_ and *A*_1g_ band peaks that are located at 384.7 cm^–1^ and 409.4 cm^–1^, respectively, with an energy difference of 24.7 cm^–1^ between the peaks for the 2-print-path sample. The similarity in the spectra for the different thicknesses is expected, as the crystallinity is associated with the flakes used for the printing. Moreover, the two Raman peaks are comparable to what has been observed in few-layer, solution-exfoliated, ALD-grown, and bulk-crystal MoS_2_ samples in terms of peak position and the energy difference between the peaks (differences of 24.6 cm^–1^ for 4–6 layers, 24 cm^–1^ for 3 layers, and 29.3 cm^–1^ for bulk crystals, respectively).^21,22^ This confirms the good crystalline quality and thickness of individual MoS_2_ flakes used for inkjet printing.

**Figure 2:**
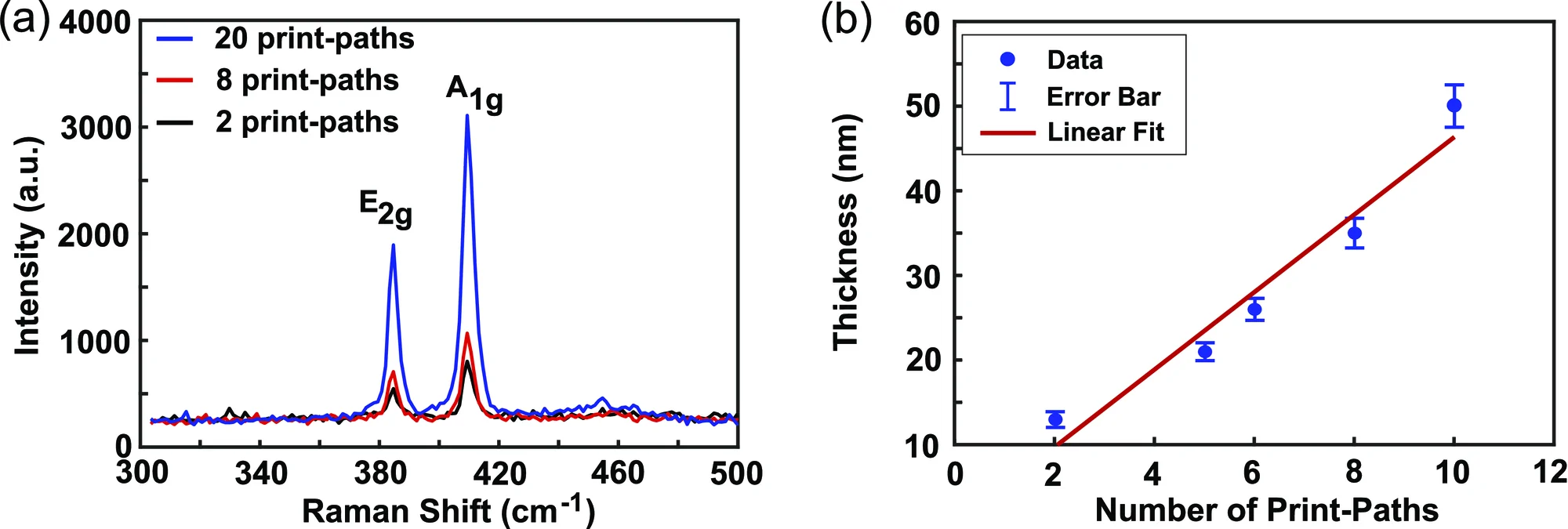
Material characterization of MoS_2_ samples printed on microscope glass slides. (a) Raman spectra of MoS_2_ samples, with the number of print-paths as labeled in the legend, showing the two characteristic *E*_2g_ and *A*_1g_ band peaks. (b) Variation of MoS_2_ sample thickness with the number of print-paths (blue circles with error bars). The red line represents a linear fit to the average layer thickness.

The thickness of the printed MoS_2_ samples was then measured using a Bruker profilometer. The profilometer was calibrated prior to each measurement, and the force setting was adjusted to 1 mg to minimize potential damage to the printed layers. The measured thickness of the MoS_2_ samples is plotted as a function of the number of print-paths in Figure 2b. The thickness of the MoS_2_ samples shows a linear relationship with increasing number of print-paths, with the thickness of one print-path ~4–5 nm. According to the Beer–Lambert law, this linearity provides compelling evidence that light passes through the material (note that the slope error of this fit is ~9%), resulting in predictable and reproducible effective thickness measurements as the number of print-paths increases.

## Simulations

### Design and Modeling

To understand the properties of the 2D material-integrated fiber devices, numerical simulations were performed to calculate the mode profiles within the D-shaped fibers, with both single-material layers and a multimaterial layered structure. The results shown in Figure 3 were obtained using the frequency-domain solver within COMSOL Multiphysics, following a configuration similar to that reported in ref. 23. Three different cases were considered: Figure 3a–c are for an MoS_2_ layer only (thickness of 30 nm, equivalent to 6 print-paths), deposited onto the flat fiber surface; Figure 3d–f are for a parylene-C layer only (1 μm thick); and Figure 3g–i are for the combined MoS_2_/parylene-C structure, as shown in the 2D sketches of the fiber cross sections in Figure 3a,d,g. A 1 μm cladding buffer was retained between the core and the polished surface, as previously discussed. The refractive indices of the fiber core and cladding were taken from standard values for SMF-28.^24^ The refractive index of the MoS_2_ layer was obtained using the bulk experimental model from Song et al.,^25^ with typical values of *n* = 4.047–4.314 across the telecom band.

**Figure 3:**
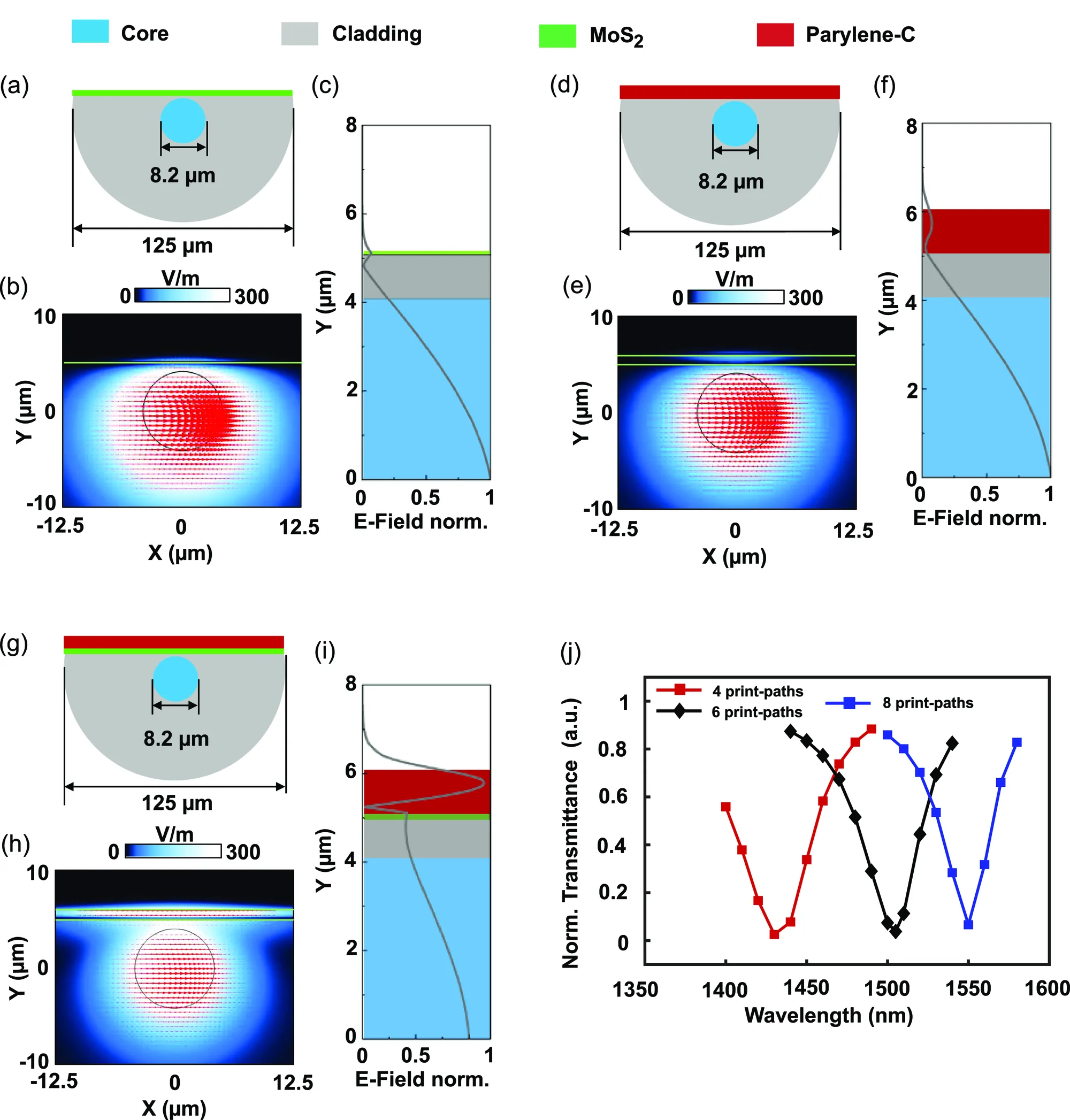
Numerical simulation results for MoS_2_-printed optical fiber. Device configuration (a, d, g), mode profile at 1.505 μm (b,e,h), and normalized electric field distribution along the *y*-axis at *x* = 0 (c, f, i). (a–c) represent a device with a 30 nm MoS_2_-printed layer only, (d-f) a device with a 1-μm-thick parylene-C-printed layer only, and (g–i) a device with both MoS_2_ and parylene-C-printed layers. (j) Simulated normalized transmittance spectra of the device in (g) with different numbers of MoS_2_ print-paths.

In this configuration, modal analysis was conducted to calculate the complex effective refractive index for the first 100 modes within the spectral range of 1.4 to 1.8 μm, with a step of 10 nm. As a starting point, the excitation wavelength was scanned until a strong interaction was observed within any of the material layers. Significantly, such strong interactions were only found to occur for TE modes in the multimaterial layered structure of Figure 3g, at wavelengths of ∼1.50 μm (Supporting Information: Simulations and measurements of the transmission of MoS_2_ only and parylene-C-only devices as functions of wavelength; see Figures S3–S5). This preferential interaction with the TE mode is similar to what has been observed in fibers integrated with CVD-produced MoS_2_ layers^14^ and is a consequence of the positive value of the imaginary part of the dynamic conductivity.^26,27^ The simulated TE mode profiles for the three cases, as calculated for the excitation wavelength of 1.505 μm, are displayed in Figure 3b,e,h. These mode profiles show how light is strongly coupled into the MoS_2_/parylene-C overlayer, but for the single-material structures, the mode remains predominantly in the core of the optical fiber. Further evidence for the differences in the mode coupling is provided by the cross section of the normalized electric field distribution, as shown in Figure 3c,f,i, where it can be seen that the guided light is only drawn out to interact strongly with the materials in the case of the multimaterial structure. Moreover, additional simulations were performed for the fiber structure of Figure 3g, where the device’s optical transmission response was calculated for the corresponding TE mode as a function of wavelength. Figure 3j shows the results for three different thicknesses of MoS_2_ layer (20, 30, and 40 nm, corresponding to 4, 6, and 8 print-paths). For the case corresponding to the 30 nm thickness used in Figure 3g, a strong wavelength-dependent absorption dip associated with resonant coupling to the materials is observed around 1.50 μm. However, these results also show how the wavelength region of the resonant coupling depends on the MoS_2_ layer thickness, with the transmittance dip moving between 1.43 and 1.55 μm. It is noted that the primary role of the parylene-C layer is to enhance the interaction with the functional 2D material and that small deviations in the thickness of the layer (±10 nm) from the target of 1 μm have a minimal effect on the position of the dip. Thus, in addition to confirming the role of the 2D material layer in supporting the resonant coupling into the multimaterial structure, the simulations show that the printed devices could be tailored to develop sensors that operate over a wide range of wavelengths by simply tuning the printed MoS_2_ thickness.

## Results and Discussion

### Optical Transmission Properties

The transmission properties of an MoS_2_-printed fiber device were subsequently investigated for both the TE- and TM-guided modes. Based on the simulation results, a fiber with a 30-nm-thick MoS_2_-printed layer (6 print-paths) and a 1-μm-thick Parylene-C capping layer was chosen, as the transmission response of this configuration falls within the range of our available laser sources. A schematic of the all-fiber experimental setup is shown in Figure 4a. Two polarized CW laser sources (Tunics T100S-HP and Agilent 8164A) with combined tunability across the wavelength range spanning 1.46–1.68 μm were used as pump sources. The polarization state of the coupled-in light was controlled using a fiber polarization controller. The output power was measured using a Thorlabs (S132C) power meter. When the TM mode was coupled into the device, the measured output power was reduced by less than a few dB for all wavelengths, and only ∼2 dB near 1500 nm, as shown in Figure 4b. The experiment was repeated for the TE mode, which, unlike the TM mode, showed a very strong interaction with the MoS_2_ layer. The transmittance spectrum of the MoS_2_-printed device for the TE mode is shown in Figure 4c. This spectrum displays a strong resonant absorption dip of ∼60 dB at 1.505 μm, in excellent agreement with the simulation results. The broader width of the measured resonance for the 6 print-path device compared with the simulation results (∼2 × larger) is most likely due to variations in the layer thickness along the 1 cm printed length.^14^ We note that confirmation of the role of the MoS_2_ thickness on the position of the resonance was obtained by characterizing a second fiber device fabricated with a 20 nm layer (4 print-paths) (Supporting Information: transmittance spectra of MoS_2_ devices with 4 and 6 print-paths (Figure S2). However, the position of the dip was at a slightly shorter wavelength (1400 nm) and had a reduced interaction (∼−46 dB vs ∼−60 dB). Thus, the 30-nm-thick MoS_2_ layer was selected for the focus of our characterization.

**Figure 4:**
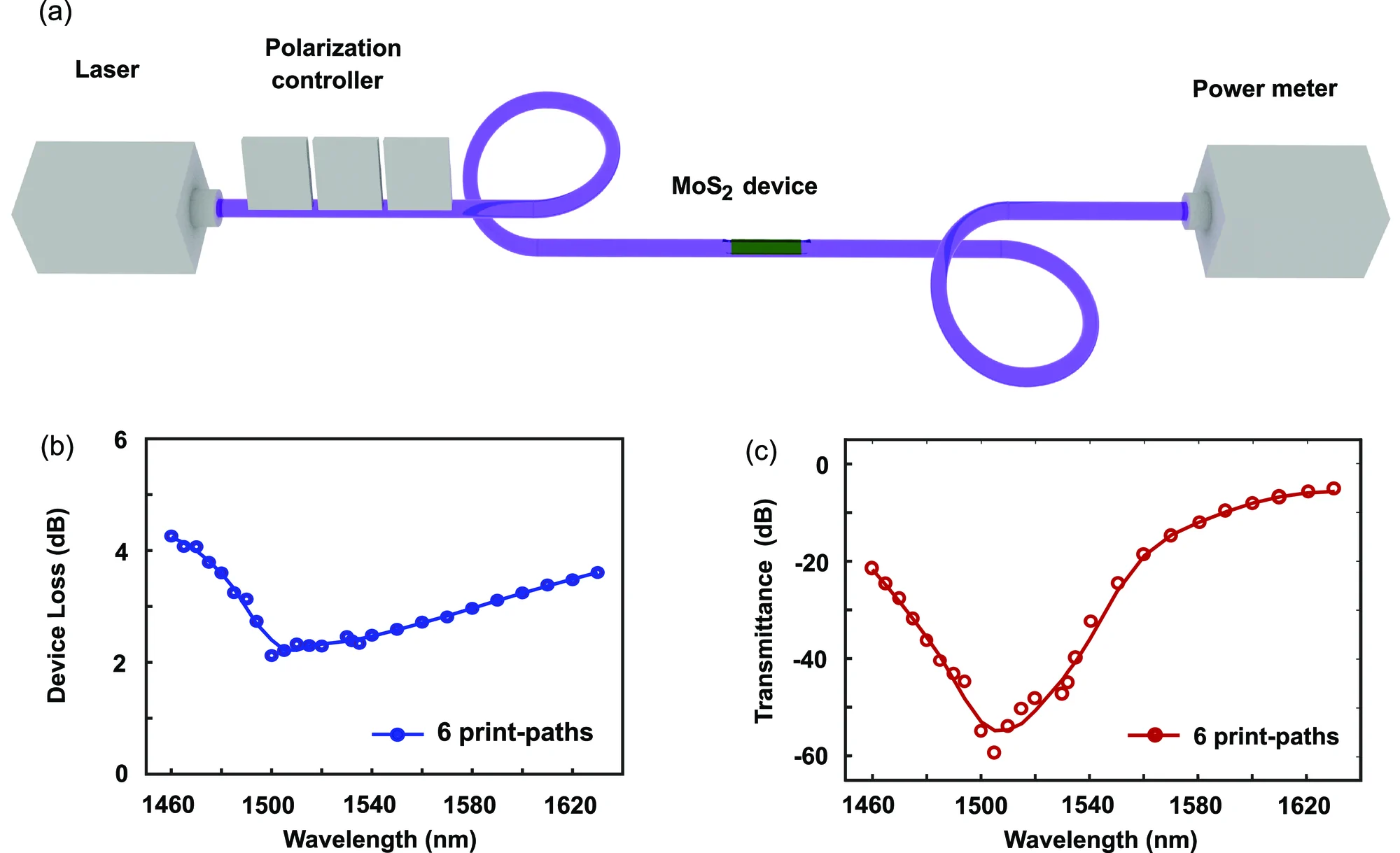
(a) Schematic of the experimental setup for the optical transmission measurements of an MoS_2_-printed fiber with 6 print-paths. (b) Device loss as a function of wavelength as measured for the TM mode (∼±5% uncertainty for each measurement). (c) Device transmittance as measured for the TE mode (∼±5% uncertainty for each measurement).

### Sensing Applications

To demonstrate the potential of MoS_2_-printed fibers for use as optical sensors, a setup to monitor ambient refractive index changes was constructed using the same 6 print-path fiber (30-nm-thick MoS_2_-printed layer and 1-μm-thick parylene-C capping layer) as characterized in Figure 4. The experimental setup is similar to that displayed in Figure 4a, but with the MoS_2_ section of the fiber placed inside a glass chamber, as shown in Figure 5a (Supporting Information: Photograph of side-polished fiber devices and experimental setup for refractive index sensing (Figure S6)). The glass chamber was filled with saline water and slightly heated to a constant temperature of ∼35 °C using a hot plate to maintain the uniformity of the saline water. Four aqueous sodium chloride (NaCl) solutions were prepared at concentrations of 0%, 10%, 20%, and 32%, corresponding to refractive indices of 1.318, 1.333, 1.351, and 1.373, respectively, limited by the solubility of the salt in deionized water.^28,29^ The transmittance spectra of the MoS_2_ fiber device in response to the four aqueous solutions are shown in Figure 5b, plotted together with the response in air. These results show a large shift in the transmittance dip from its initial position at 1.505 μm (*n* = 1) to 1.651 μm (*n* = 1.318) due to the large increase in the refractive index when the device is exposed to pure water (0% salt). Then, as the refractive index of the aqueous salt solutions increases further, the position of the transmittance dip shifts toward even longer wavelengths, reaching 1.68 μm for a maximum obtainable refractive index of 1.373, corresponding to 32% salt water. It is noted that the sensor recovers to its original state when washed and dried (Supporting Information: MoS_2_ device transmittance before and after saline water test (Figure S7)).

**Figure 5:**
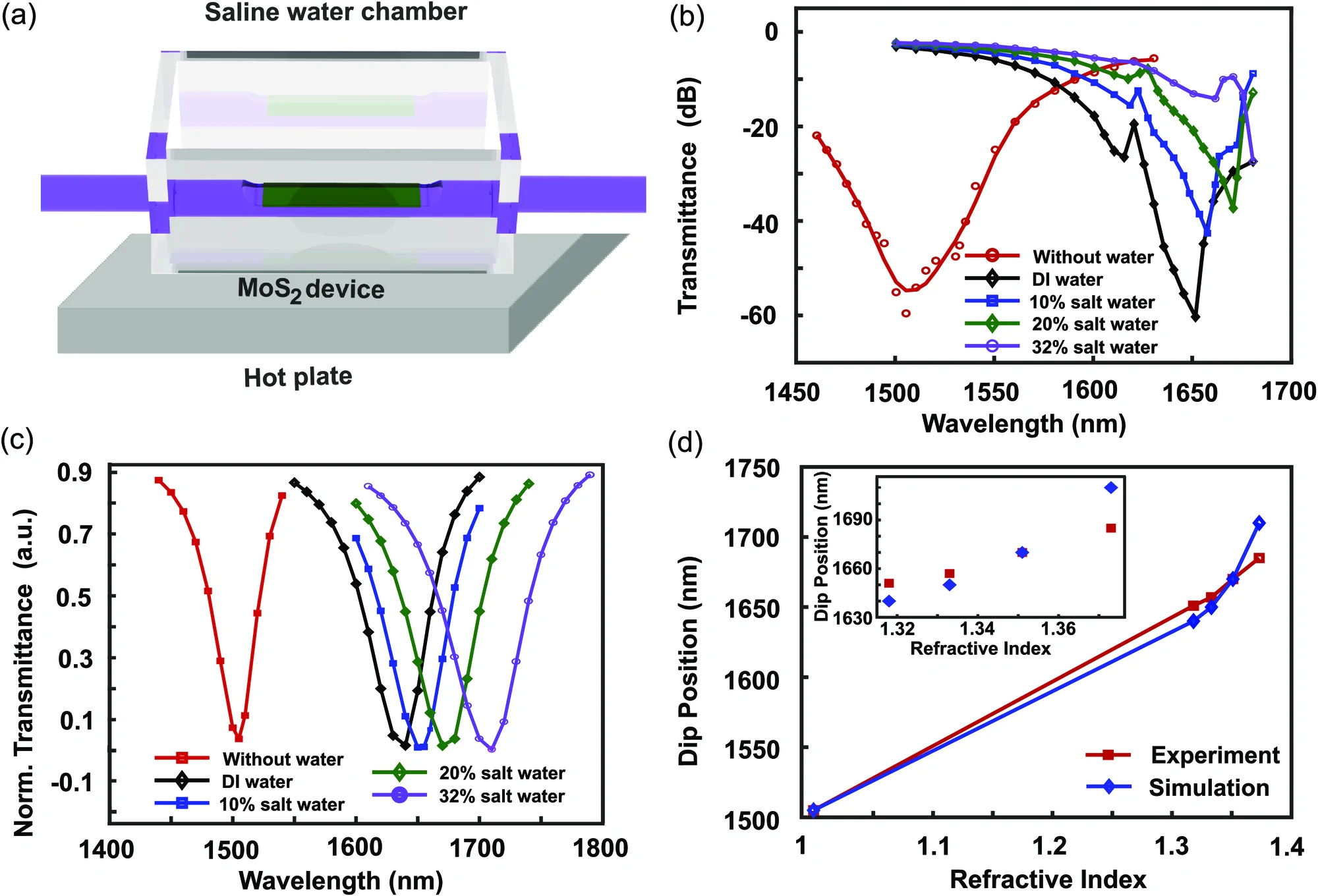
Characterization of an MoS_2_ fiber with 6 print-paths as a refractive index sensor. (a) Experimental setup for refractive index sensing. (b) Experimental transmittance spectrum of the fiber device while submerged in different aqueous sodium chloride solutions (∼±5% uncertainty for each measurement). (c) Simulated normalized transmittance spectra of the fiber device with different aqueous sodium chloride solutions. (d) Variation in the position of the transmittance dip in the MoS_2_-printed fiber as a function of refractive index, as determined by experiments (red squares) and simulations (blue diamonds).

For comparison, the numerical results in Figure 3j for the 6 print-path device were extended to simulate the role of the salt solution on top of the MoS_2_/parylene-C layer. Figure 5c shows the calculated normalized transmittance spectra of the fiber device when surrounded by aqueous salt solutions corresponding to the same four concentrations. It is important to note that, as the numerical model only accounts for the loss of the MoS_2_ material, which is relatively flat over the telecom band, and not the additional losses due to the increasing concentration of the saline solutions, the depths of the simulated transmittance dips are relatively unchanged. As before, we attribute the broader experimental absorption dips to variations in the MoS_2_ layer thickness. However, there is good agreement between the dip positions, as shown in Figure 5d where the experimental and simulation results are compared directly. From the slope of the dip position as a function of refractive index unit (RIU), we calculate the sensitivity of our fiber sensor to be ∼ 1200 nm/RIU. Although this value is comparable in magnitude to other experimental fiber sensors using MoS_2_ layers, it is lower than the best devices that make use of deposited materials and more complex material structures.^13,30^ However, the sensitivity could be optimized by tuning the ratio of the MoS_2_ and parylene-C layers, and potentially including more printed material layers.^31^ We note that although standard SMF fibers were employed in our studies, owing to the strong polarization dependence of the active sensing region, polarization-maintaining fibers would need to be used for the construction of large-scale or remote sensing applications. Nevertheless, the good sensitivity, coupled with the simple fabrication of the inkjet-printed MoS_2_ fibers, motivates further exploration of their use in environmental gas or temperature sensing.

## Conclusion

We have used numerical simulations and experiments to demonstrate a low-loss and flexible MoS_2_ integrated fiber device. The MoS_2_ was inkjet-printed on D-shaped single-mode fibers using a low-cost and scalable technique that produced materials with good crystallinity. A MoS_2_ device with a thickness of 30 nm was found to exhibit a strong wavelength-dependent transmission spectrum associated with resonant coupling to the 2D material, with an absorption dip of ∼60 dB at 1.505 μm. The position of the transmittance dip agrees well with simulation results for the two-dimensional structure, with the modeling showing that the dip position can be tuned by varying the 2D material layer thickness. Furthermore, a refractive index sensor based on the MoS_2_-integrated fiber has been experimentally demonstrated, with the device performance agreeing well with the numerical predictions. The results highlight the potential of inkjet-printed MoS_2_ fibers for the development of large-scale sensing networks and environmental monitoring.

## Supplementary Material



## Data Availability

The data that support the findings of this study are openly available at the University of Southampton at 10.5258/SOTON/D4010

## Notes

Polished fiber surface characterization; transmittance spectra of MoS_2_ devices with 4 and 6 print-paths; transmittance spectra of MoS_2_ only devices with 6, 100, and 300 print-paths without any parylene-C layer; simulated transmission response of devices with different parylene-C thickness (without MoS_2_ layers); simulated transmission response of devices with different MoS_2_ thickness (without a parylene-C layer); photograph of side-polished fiber devices and the experimental setup for refractive index sensing; MoS_2_ device transmittance before and after the saline water test (PDF)
