# Supplementary material for: Inkjet-Printed MoS2 D‑Shaped Fiber for Resonance-Enhanced Optical Sensing
Source: ACS Photonics. 2026 Sep 30;13(20):6076–83. doi: 10.1021/acsphotonics.6c01158 (PMC13693495; doi:10.1021/acsphotonics.6c01158)
Supplement: Supplementary file 1 [file ph6c01158_si_001.pdf]

Supporting Information

Inkjet-printed MoS_2 D-shaped fiber for resonance enhanced optical sensing

Amar N. Ghosh*,†, Mingfei Xiao‡, Jinrui Chen‡, Victor Pacheco-Pena¶, Meng Huang†, Noel Healy¶, Tawfique Hasan‡, and Anna C. Peacock†

†Optoelectronics Research Centre, University of Southampton, Southampton SO17 1BJ, UK.

‡ Department of Engineering, University of Cambridge, Cambridge, CB3 0FA, UK

¶School of Mathematics, Statistics, and Physics, Newcastle University, Newcastle upon Tyne, NE1 7RU, UK

*Corresponding author: A.N.Ghosh@soton.ac.uk

This document provides supporting information to "Inkjet-printed MoS_2 D-shaped fiber for resonance enhanced optical sensing". The figures are intended to enhance the readers understanding of some of the experimental methods used in the paper. **© 2026 ACS Publications**

Note 1: Side polished fiber surface characterisation

SEM image and ZeScope 3D optical profiler measurements of a polished fiber surface are shown in **Fig. S1**. These measurements confirm the surface smoothness (RMS roughness of ~ 0.8 nm) and the flatness (a longitudinal angle of only 0.04° was measured.

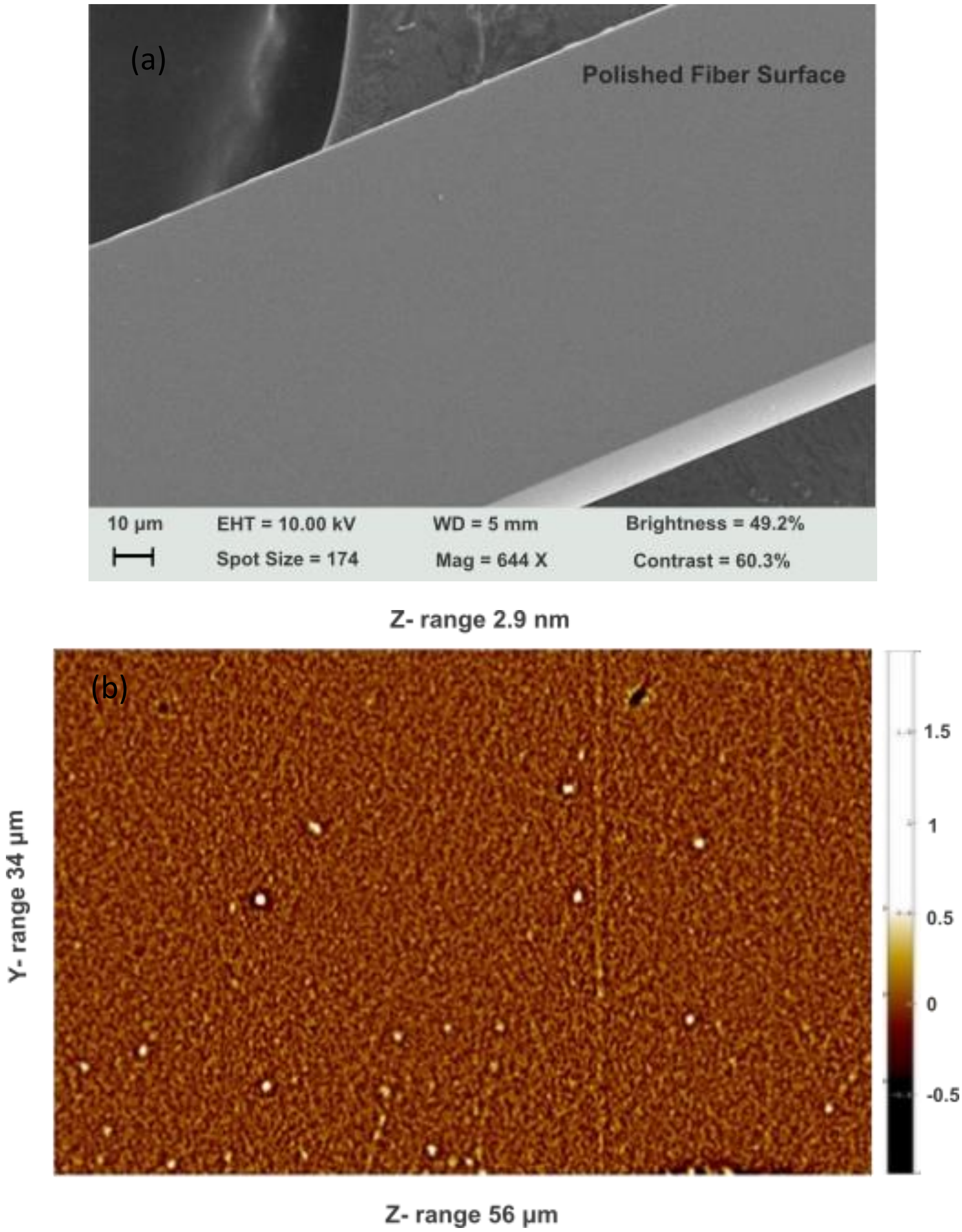

Figure S1. Polished fibre surface characterisation. (a) SEM image of the polished surface. (b) The surface quality was measured with a ZeScope 3D optical profiler. Surface roughness: RMS = 0.8 nm, with max features ~ 1.5 nm. Flatness: a longitudinal angle of 0.04 ° was measured.

Note 2: Transmission of MoS_2 devices with different thickness

Two fibre samples were prepared with different MoS_2 thicknesses (four and six print-paths) and with the same parylene-C thicknesses. The transmission properties for TE modes are compared in the following Fig. S2, showing a shift in the resonance position for the change in MoS_2 thickness, in agreement with the predictions of the simulation. The predicted and measured transmittance dip positions are 1430 nm and 1400 nm, respectively for four print-paths device and 1505 nm and 1505 nm, respectively for six print-paths devices.

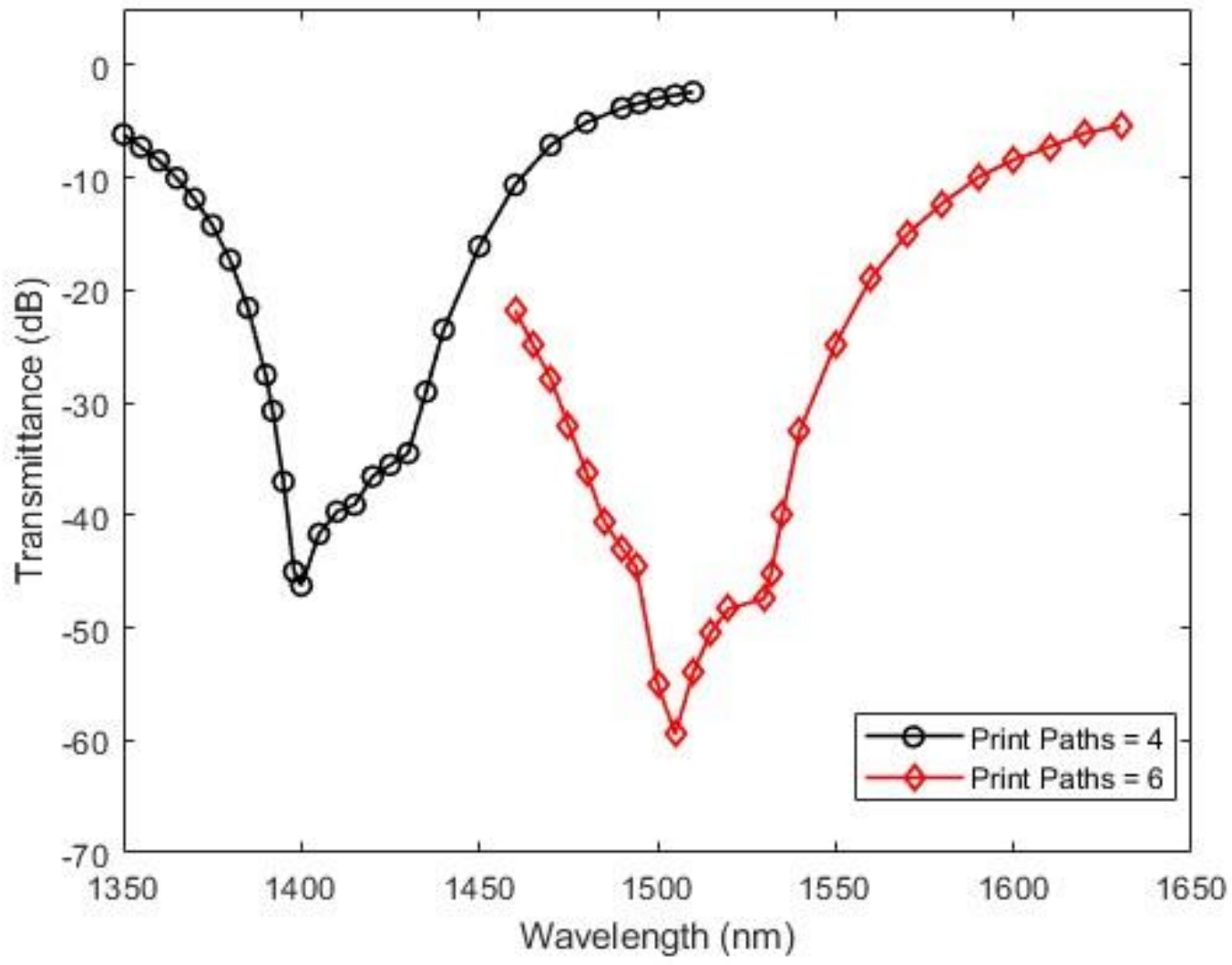

Figure S2: Transmittance spectra of MoS_2 devices with 4 and 6 print-paths.

Note 3: Measurements and simulations of the transmission of MoS_2 only and parylene-C only devices

Several fiber samples were also fabricated with different MoS_2 thicknesses (6, 100, and 300 print-paths) without any parylene-C layer. The optical responses for these samples were relatively flat, representative of a constant loss with no transmittance dip as shown in Fig. S3.

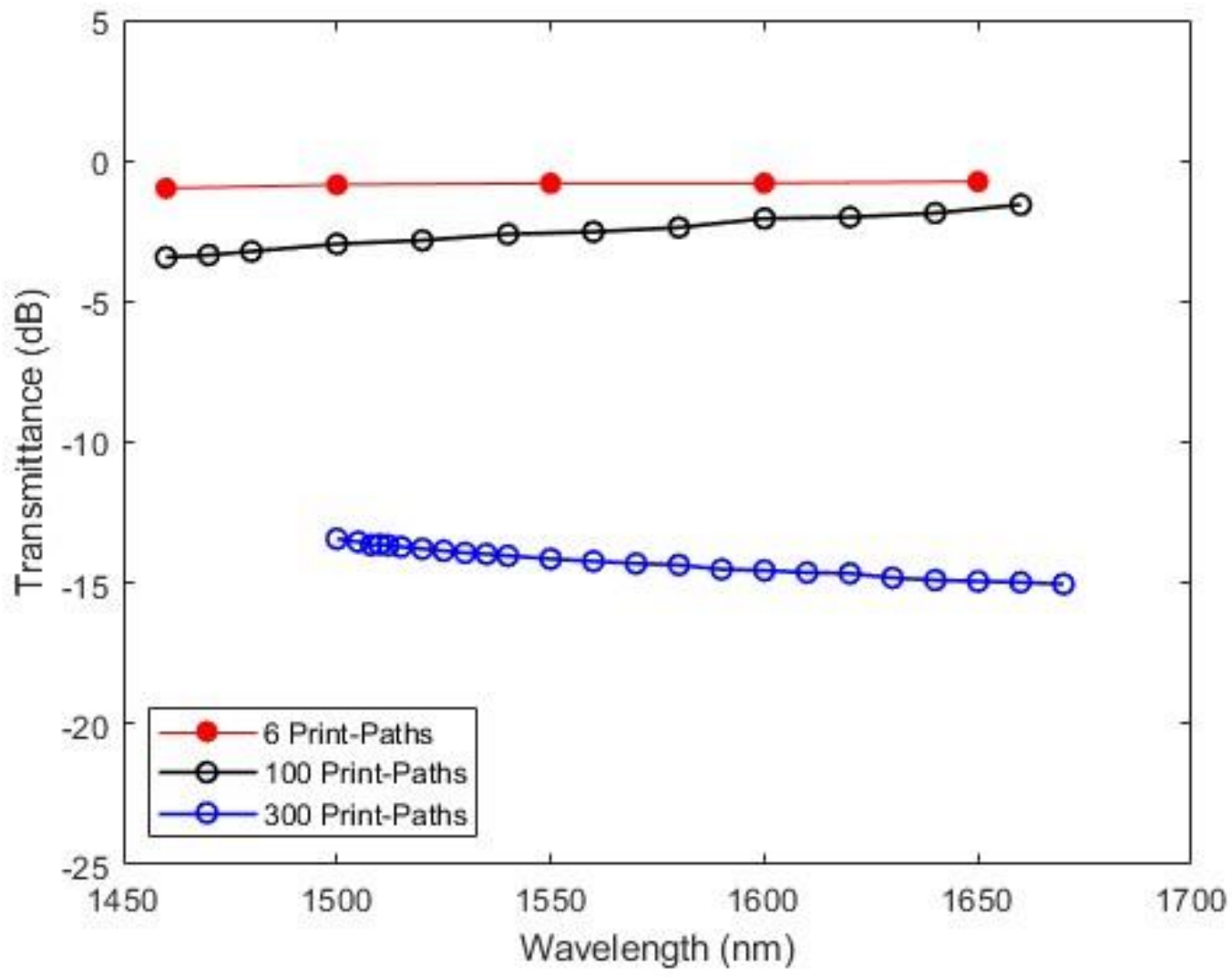

Figure S3: Transmittance spectra of MoS_2 only devices with 6, 100, and 300 print-paths without any parylene-C layer.

Simulations were conducted of fiber structures with different parylene-C thickness (without MoS_2 layer) to confirm that such structures do not show any evidence of wavelength dependent light–matter interaction, as shown in Fig. S4. Note that the parylene-C layer was assumed to be lossless in this simulation. Further simulations were also conducted of structures with different MoS_2 thickness (without any parylene-C layer) and their transmission as a function of wavelength are shown in Fig. S5.

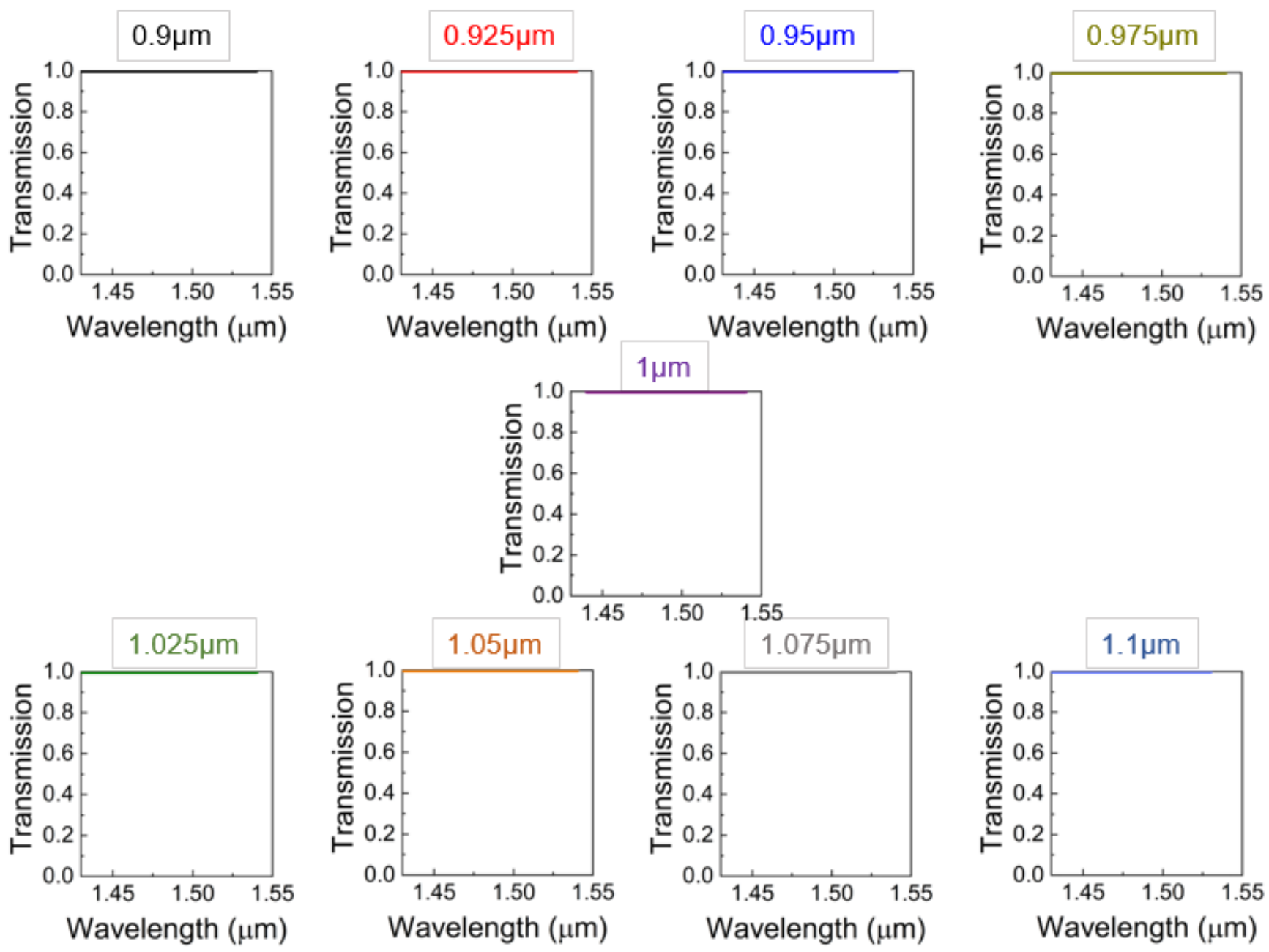

Fig. S4: Simulated transmission response of devices with different parylene-C thickness (without MoS_2 layers).

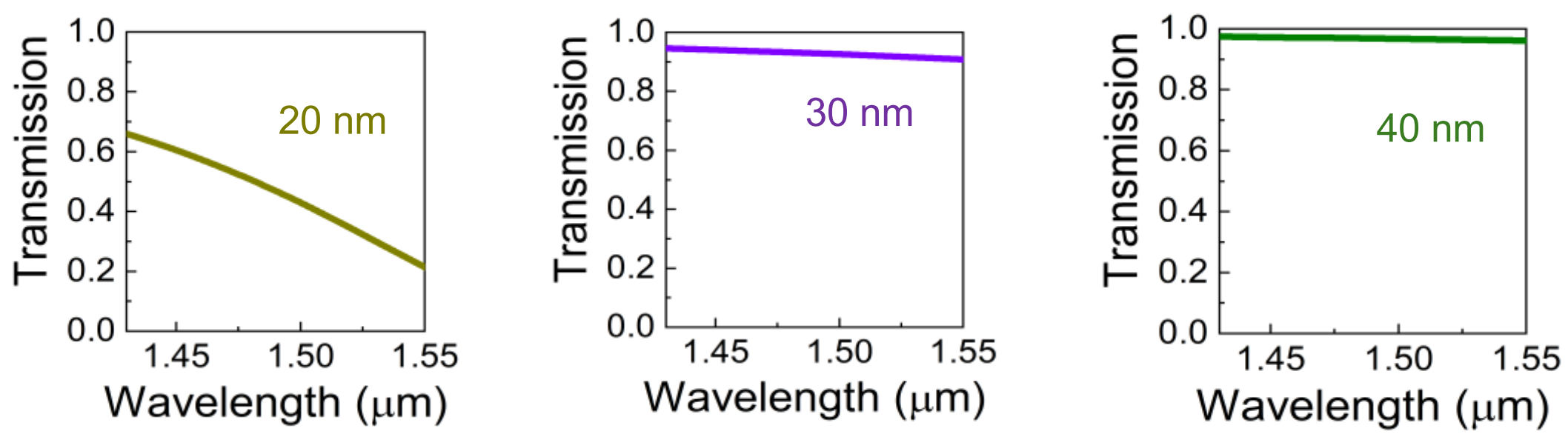

Figure S5: Simulated transmission response of devices with different MoS_2 thickness (without parylene-C layer).

Note 4: Photograph of side polished fiber devices and experimental setup for refractive index sensing

For better visualization of the fiber device and sensing setup, Fig. S6 shows a photograph of the chamber for the sensing setup, a photograph of the MoS_2 printed side polished fiber, and a microscope image of the polished fiber surface.

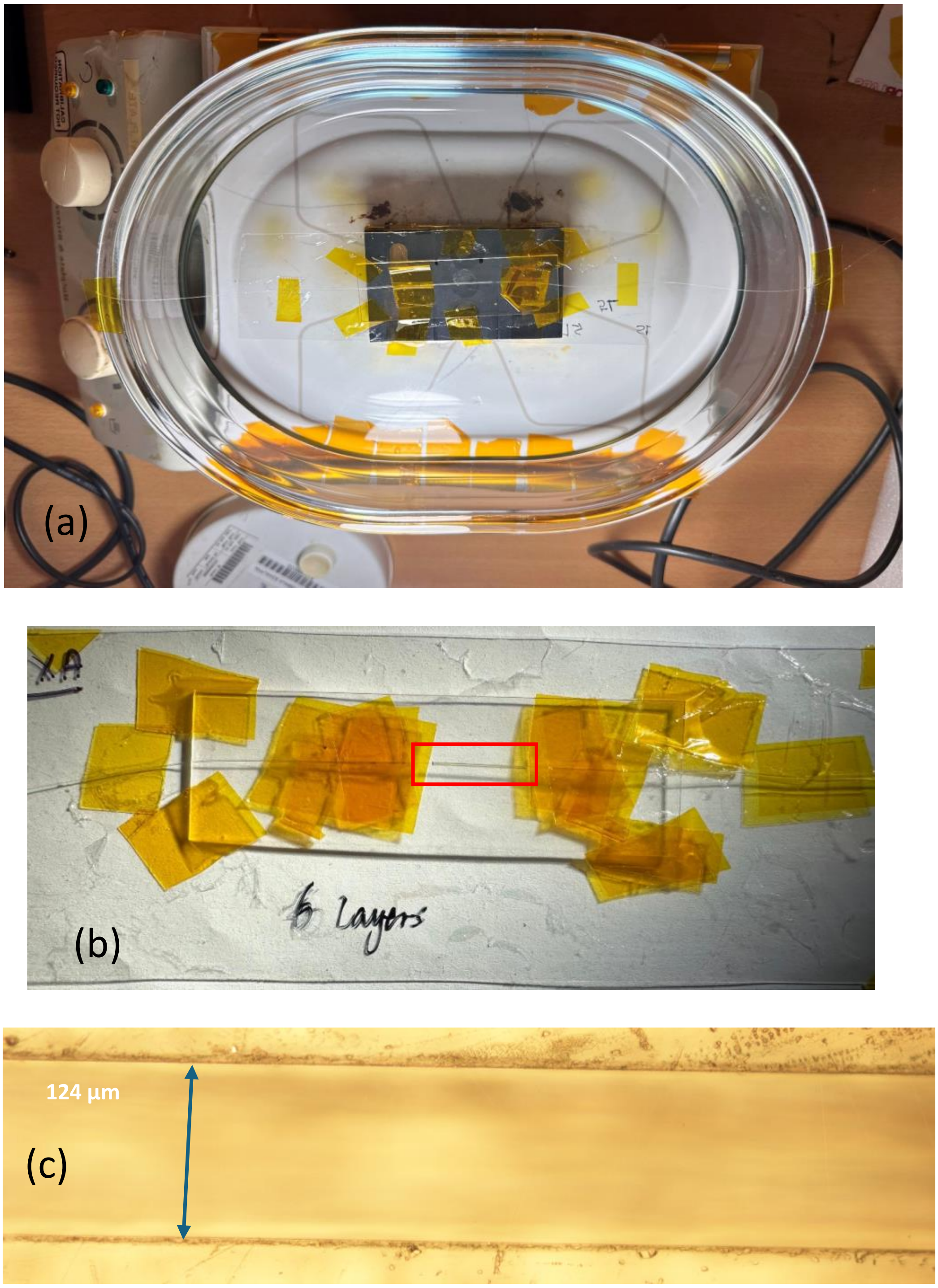

Figure S6: (a) Photograph of the experimental setup for refractive index sensing. This image represents the same figure reported as Figure 5(a) in the manuscript. (b) Photograph of MoS_2 printed device where the printed area is highlighted by red box. (c) Optical microscope image of the polished surface.

Note 5: Reversibility test of refractive index sensor

To demonstrate the repeatability and reversibility of our refractive index sensor, we have compared the device's transmittance response before and after the saline water test as shown below in Fig.S7.

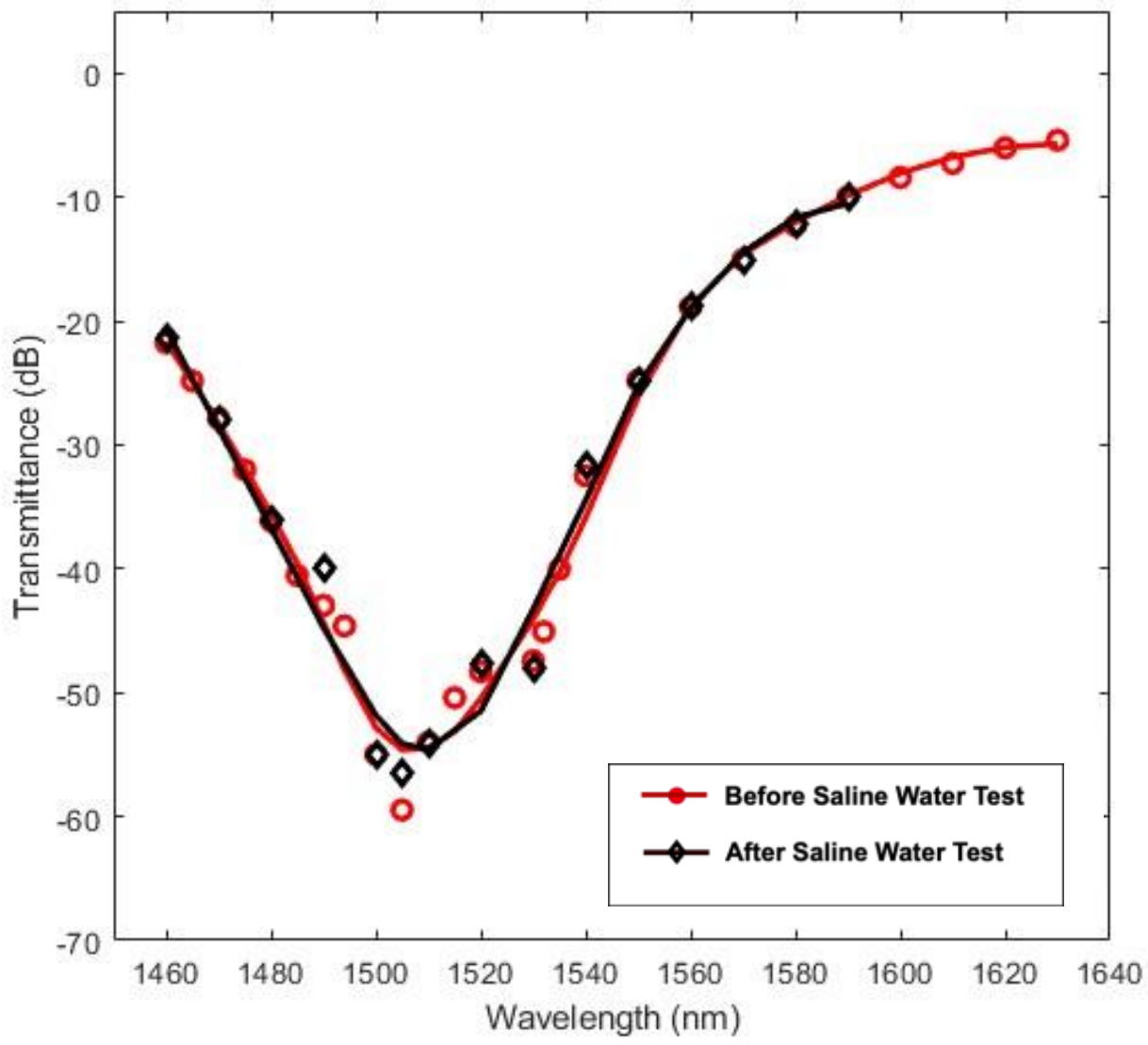

Figure S7. MoS_2 device transmittance before (red) and after (black) the saline water test.
